# Epidemiologic, Clinical and Mycological Profile of Onychomycosis in the Hospital Setting in Benin

**DOI:** 10.1155/2024/1056753

**Published:** 2024-10-12

**Authors:** Fabrice Akpadjan, Yolande Sissinto Savi De Tove, Aminath Fèmie Tidjani, Cordule Balola, Laura Dotsop, Bérénice Degboe, Nadège Agbessi, Christiane Koudoukpo, Hugues Adegbidi, Félix Atadokpede, Florencia do Ango-Padonou

**Affiliations:** ^1^Service de Dermatologie-Vénérologie, Centre de Dépistage et de Traitement de L'Ulcère de Buruli d'Allada, Faculté des Sciences de La Santé-Université d'Abomey-Calavi, Cotonou, Benin; ^2^Laboratoire de Parasitologie-Mycologie, Faculté des Sciences de La Santé-Université d'Abomey-Calavi, Cotonou, Benin; ^3^Unité d'Enseignement et de Recherche en Pharmacie, Faculté des Sciences de La Santé-Université d'Abomey-Calavi, Cotonou, Benin; ^4^Service de Dermatologie-Vénérologie, Centre National Hospitalier et Universitaire Hubert Koutoukou Maga de Cotonou, Faculté des Sciences de La Santé-Université d'Abomey-Calavi, Cotonou, Benin; ^5^Service de Dermatologie-Vénérologie, Centre Hospitalier Universitaire Département du Borgou-Alibori, Faculté de Médecine de L'Université de Parakou, Parakou, Benin

**Keywords:** Benin, *Candida albicans*, fingernails, onychomycosis

## Abstract

**Introduction:** Onychomycosis accounts for 50% of nail disorders and remains one of the most frequent reasons for consultation in dermatology. Several factors favour the development of onychomycosis, such as age, morphological abnormalities of the nails, immunodeficiency and certain underlying pathologies. The aim was to study the epidemiological and diagnostic profile of onychomycosis in patients treated in the dermatology and venerology department of the Benin referral hospital.

**Patients and Methods:** This was a retrospective study conducted from 2003 to 2022, covering patients treated for onychomycosis in the Dermatology–Venerology University Clinic at the National University Hospital Center Hubert Koutoukou Maga (NUHC-HKM) in Cotonou.

**Results:** During the study period, 389 new patients were seen for onychopathy, 301 of whom had a clinical suspicion of onychomycosis. Of these, 128 were confirmed by mycological examination, giving a hospital frequency of onychomycosis of 32.90% compared with onychopathy. The mean age was 44.93 ± 12.50 years; the patients were predominantly female, with a sex ratio (M/F) of 0.41. Aesthetic complaints (76.47%) were the most frequent reason for consultation, and the nail of the hand was the most common lesion location. Melanonychia was the most frequent morphological anomaly, and distolateral subungual onychomycosis was the most frequent clinical form. Yeasts (86.92%) were the most isolated group of fungi, followed, respectively, by moulds (08.44%) and dermatophytes (04.64%). *Candida albicans* (21.52%) was the most common species.

**Conclusion:** Onychomycosis is relatively uncommon in hospitals in Benin. This is underestimated because most patients with a clinical suspicion of onychomycosis do not undergo a mycological examination before being put on antifungal treatment.

## 1. Introduction

Onychomycosis is a nail infection caused by a fungus. It accounts for 50% of nail disorders [1] and remains one of the most common reasons for consultations in dermatology. The prevalence of this condition in the general population ranges from 2% to 26% and is constantly increasing; particularly among immunodeficient patients, such as those with HIV/AIDS [2, 3]. Several factors promote the development of onychomycosis, such as age, genetics, nail morphological anomalies, immunodeficiency and certain underlying pathologies [4, 5]. Diagnosis is most often clinically suspected when there is a change in nail colour or when it becomes fragile. Diagnostic confirmation is provided by mycological examination performed on a nail fragment. Few studies on onychomycosis in a hospital setting have been conducted in Benin. The aim of this study was to investigate the epidemiological and diagnostic profile of onychomycosis among patients treated at the University Clinic of Dermatology–Venereology of the National University Hospital Center Hubert Koutoukou Maga (NUHC-HKM) in Cotonou from 2003 to 2022.

## 2. Patients and Methods

This was a retrospective descriptive study conducted from January 2003 to December 2022, involving the records of patients seen for onychomycosis at the University Clinic of Dermatology–Venereology of NUHC-HKM in Cotonou. The study population consisted of patients who consulted for nail pathology at the University Clinic of Dermatology–Venereology and who had undergone a nail sampling for mycological examination during the defined study period and met the selection criteria. Mycological diagnosis was carried out by direct examination and culture. If positive for moulds, a second culture was taken for confirmation. Data were collected from consultation registers and patient files from the Dermatology–Venereology unit. They were recorded using the KoboCollect application. Data analysis was performed using R software Version 4.0.3. Qualitative variables were described in terms of frequency and percentage and quantitative variables in the median with interquartile range due to the usual asymmetry of their distributions at the Shapiro–Wilk test (*p* < 0.05). Patient anonymity was maintained.

## 3. Results

### 3.1. Hospital Frequency

During the study period, 23,806 new patients were seen in consultation at the University Clinic of Dermatology–Venereology at NUHC-HKM in Cotonou (Benin). Among them, 389 new patients consulted for a nail pathology of which 301 had a clinical suspicion of onychomycosis, and 128 of them had confirmation through mycological examination. The overall frequency of onychomycosis in the said service was, therefore, 0.54%, and the hospital frequency of onychomycosis in relation to nail pathologies was 32.90%.

### 3.2. Sociodemographic Characteristics of the Population

- The average age was 44.93 ± 12.50 years with extremes from 3 to 84 years.
- There was a female predominance with a sex ratio (M/F) of 0.41
- The majority of patients resided in urban areas (87.69%) and were civil servants. Only 4 patients were strictly housewives.

### 3.3. Clinical Data

**Figure 1:**
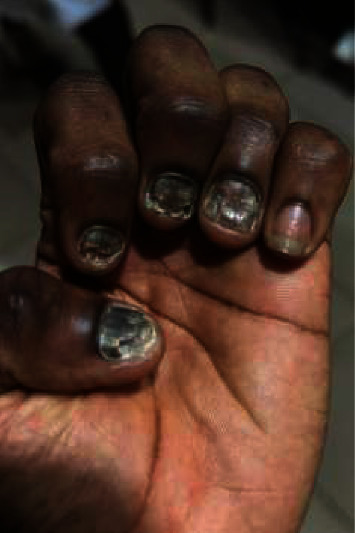
Onychomycosis with onychodystrophy and onycholysis of the first four nails of the left hand.

**Figure 2:**
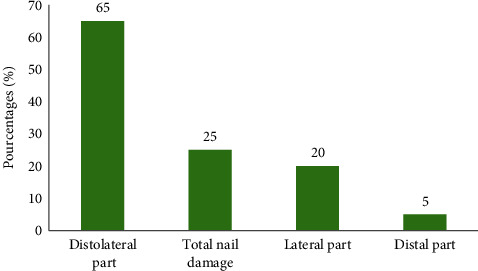
Distribution of patients with onychomycosis at the University Clinic of Dermatology–Venerology of NUHC-HKM = National University Hospital Center Hubert Koutoukou Maga in Cotonou from 2003 to 2022 according to the clinical presentation (location) of the onychomycosis.

**Figure 3:**
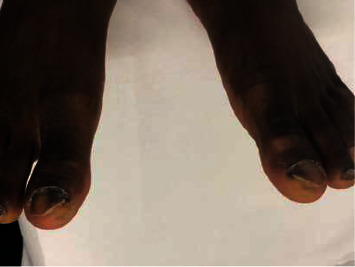
Distolateral subungual onychomycosis of the nails of the two big toes with melanonychia and onycholysis due to *Candida parapsilosis*.

- Aesthetic discomfort was the main reason for consultation among most patients with onychomycosis (76.47%)
- The lesions were primarily located on the fingernails (Table 1).
- Melanonychia was the most observed clinical aspect of the nail (Table 2), followed by onycholysis (Figure 1).
- Distolateral subungual onychomycosis was the most common clinical form (Figures 2 and 3)

### 3.4. Mycological Data

- Among the 301 patients with a clinical suspicion of onychomycosis, 325 samples were taken from the fingernails and/or toenails.
- Out of the 325 samples, culture was positive on 237 samples, corresponding to 128 patients. Several samples were taken from the same patient.
- Three groups of fungi were isolated in these cultures with a predominance of yeasts (Table 3). Some patients had an association of different groups of fungi.
- According to the location of the different champion species, it was found that the yeast (86.92%) predominated on the hands, while the dermatophyte predominated on the feet.
- *Candida albicans* (21.52%) dominated the yeast group, while *Aspergillus niger* (3.79%) was the most commonly found mould. As for dermatophytes, they were dominated by *Trichophyton rubrum* (4.22%) (Table 4).

## 4. Discussion

### 4.1. Sociodemographic Data

- The average age of patients in our study was 44.93 ± 12.50 years. Our results align with those of Akakpo, Zoua and Téclessou [6] in Togo who found an average age of 43.03 ± 14.5 years and Kouotou et al. [7] in Cameroon who reported an average age of 40.7 ± 13.1 years. However, on the island of Crete in Greece [8], a 12-year epidemiological study found an average age of 53 years. Similarly, another study by Papini et al. [9] in Italy found an average age of 60 years. This difference could be due to the younger population in Africa compared with developed countries where life expectancy is higher.
- Women were more affected with a sex ratio (M/F) of 0.41. This result is consistent with that of A. Tosti et al. [10] who also found a female predominance with a sex ratio of 0.4. Similarly, Ngaba et al. [11] in Cameroon reported a sample consisting mostly of women (69.4%). This predominance may be explained by the functional discomfort and aesthetic concerns, more frequently expressed by women [12, 13].
- Socioprofessionally, only 4 patients were housewives. This low representation can be explained by financial constraints related to consulting in dermatology and performing mycological examinations in our context by this economically disadvantaged social layer. These high costs limit access to care for low-income individuals and consequently lead to an underestimation of certain socioprofessional groups most at risk of onychomycosis.

### 4.2. Clinical Data

The majority of patients (76.47%) cited aesthetic discomfort as the reason for consultation. The discomfort or aesthetic concern is one of the major consequences of onychomycosis, making this condition not only a cosmetic problem but also a significant health issue. Our results corroborate those of the existing literature [14, 15].

Regarding the location of the nail abnormality, hands were most frequently affected (65.52%). This predominance of the hands can be explained by their physiological and social function in daily gestures, social interactions and cosmetic care and also by the female predominance in our study. These results are consistent with other studies conducted in Togo and Tunisia [6, 16], which also showed a predominance of hand involvement.

As concerns the clinical presentation, distolateral subungual onychomycosis was the most frequent (65%). This finding is aligned with several other studies, including those of Ngaba et al. [11], Seck et al. in Senegal [17] and many other authors, where distolateral subungual onychomycosis also predominated.

### 4.3. Mycological Data

The yeast group (86.92%) predominated on the hands, and the most commonly found species was *Candida albicans* (21.52%). The dermatophyte group predominated on the feet, and *Trichophyton rubrum* was the most found species. These results are consistent with those of Konaté et al. [10], who identified yeasts, predominantly *Candida tropicalis* (36.4%) and *Candida albicans* (30.3%), and dermatophytes, predominantly *Trichophyton rubrum*. However, this differs from Greek [18], French [4, 19] and Iranian [20] studies where dermatophytes were the primary group of isolated fungi. This could be due to climatic differences between these regions of the world.

## 5. Conclusion

At the end of this study, it is important to note the low hospital frequency of documented onychomycoses in a hospital setting in Benin. This is likely underestimated because most patients with a clinical suspicion of onychomycosis do not undergo mycological examination for diagnostic confirmation before being treated with antifungal agents. The most frequently found pathogen in culture is *Candida albicans*, with a predilection for the nails of the hands. A prospective study will help us to identify the factors associated with the occurrence of onychomycosis in our country.

## Figures and Tables

**Table 1:** Distribution of patients with onychomycosis at the University Clinic of Dermatology–Venerology of NUHC-HKM in Cotonou from 2003 to 2022 according to the site of lesions.

	Numbers (*n*)	Proportions (%)
Fingers	121	59.02
Toes	52	25.37
Fingers and toes	32	15.61
Total	205	100.00

**Table 2:** Distribution of patients with onychomycosis at the University Clinic of Dermatology–Venerology of NUHC-HKM in Cotonou from 2003 to 2022 according to the morphological abnormalities of the nails.

	Number (*n*)	Proportions (%)
Melanonychia	39	24.37
Onycholysis	25	15.62
Trachyonychia	15	09.37
Leuconychia	14	08.75
Onychodystrophy	12	7.5
Pachyonychia	11	06.87
Subungual hyperkeratosis	10	06.25
Xanthonychia	5	03.12
Unspecified nail dyschromia	4	02.50
Chloronychia	2	01.25
Total	160	100

**Table 3:** Distribution of the 237 culture positive samples according the fungus groups found in patients with onychomycosis at the University Clinic of Dermatology–Venerology of NUHC-HKM in Cotonou from 2003 to 2022.

	Numbers (*n*)	Proportions (%)
Yeast	206	86.92
Moulds	20	08.44
Dermatophytes	11	04.64
Total	237	100

**Table 4:** Distribution of the main fungal species isolated in the 237 culture positive samples in patients with onychomycosis at the University Clinic of Dermatology–Venerology of NUHC-HKM in Cotonou from 2003 to 2022.

	Number	Proportions (%)
Yeast		
*Candida albicans*	51	21.52
*Candida parapsilosis*	46	19.41
*Candida famata*	15	6.32
*Candida sake*	12	5.06
*Candida sp*	12	5.06
*Candida tropicalis*	9	3.79
*Candida guilliermondii*	8	3.37
Moulds		
*Aspergillus niger*	9	3.79
*Aspergillus candidus*	3	1.27
*Aspergillus fumigatus*	3	1.27
*Trichosporon asahii*	8	3.37
*Trichosporon spp*	3	1.27
*Fusarium spp*	3	1.27
*Fusarium solani*	3	1.27
Dermatophytes		
*Trichophyton rubrum*	10	4.22
*Trichophyton interdigitale*	3	1.27
*Trichophyton soudanense*	3	1.27
*Trichophyton tonsurans*	2	0.84

## Data Availability

The data that support the findings of this study are available from the corresponding author upon reasonable request.
